# Association of *CATSPER1*, *SPATA16* and *TEX11* genes polymorphism with idiopathic azoospermia and oligospermia risk in Iranian population

**DOI:** 10.1186/s12920-022-01197-w

**Published:** 2022-03-05

**Authors:** Mohammadreza Behvarz, Seyyed Ali Rahmani, Elham Siasi Torbati, Shahla Danaei Mehrabad, Maryam Bikhof Torbati

**Affiliations:** 1 Department of Genetics, Faculty of Biological Science, North Tehran Branch, Islamic Azad University, Tehran, Iran; 2 Department of Medical Genetics, School of Medicine, Tabriz University of Medical Sciences, Tabriz, Iran; 3 Department of Gynecology, Eastern Azerbaijan ACECR ART Center, Eastern Azerbaijan Branch of ACECR, Tabriz, Iran; 4 Department of Biology, Yadegar-e-Imam Khomeini (RAH) Shahr-e-Rey Branch, Islamic Azad University, Tehran, Iran

**Keywords:** Male infertility, *CATSPER1*, *SPATA16*, *TEX11*, Polymorphism

## Abstract

**Background:**

Male infertility is a heterogeneous disease which can occur due to spermatogenesis defects. The idiopathic azoospermia and oligospermia are the common cause of male infertility with unknown underlying molecular mechanisms. The aim of this study was to investigate association of idiopathic azoospermia and oligospermia with single-nucleotide polymorphisms of *CATSPER1*, *SPATA16* and *TEX11* genes in Iranian-Azeri men.

**Methods:**

In this case–control study, we recruited 100 infertile men (case group) and 100 fertile men (control group) from Azeri population in north western provinces, Iran, population. The genomic DNA was extracted using a proteinase K method from peripheral blood leukocytes. The genotypes analysis was conducted using tetra-primer amplification refractory mutation system-polymerase chain reaction method. The obtained data were analyzed by statistical software.

**Results:**

We found a significant difference in the frequencies of heterozygote AB and mutant homozygote BB genotypes in the *CATSPER1* (rs2845570) gene polymorphism between patients and healthy controls (*p* < 0.05). Moreover, we observed a significant difference in the frequencies of heterozygote BA genotype in the *SPATA16* (rs1515442) gene polymorphism between patients and healthy controls (*p* < 0.05). However, no significant difference was found in genotypes distribution of case and control groups in the *TEX11* (rs143246552) gene polymorphism.

**Conclusion:**

Our finding showed that the *CATSPER1* (rs2845570) and *SPATA16* (rs1515442) genes polymorphism may play an important role in idiopathic azoospermia and oligospermia in Iranian Azeri population. However, more extensive studies with larger sample sizes from different ethnic origins are essential for access more accurate results.

## Background

Infertility is defined by the world health organization as a disorder of reproductive system that is one of the important problems in 10–15% of couple worldwide, which male infertility factors are responsible for 20–30% of all human infertility cases [1, 2]. One of the common cause of the male infertility is defects in quality and quantity of sperm, which can occur by effects of genetic variants and environmental factors [3, 4]. The idiopathic azoospermia and oligospermia are common spermatogenesis disorders in men, and is observed in a high proportion of infertile men [5]. However, the underlying molecular mechanisms of the spermatogenesis defects remain unknown [6, 7].

Evidences suggested that male infertility can occur due to mutations or single-nucleotide polymorphisms (SNPs) in various genes [8, 9]. Recently, much studies have been performed to investigate diverse mutations and SNPs in candidate genes involved in male infertility [10, 11]. Miscellaneous variants in *CATSPER1*, *SPATA16*, and *TEX11* genes are identified that play important roles in pathogenesis of sperm morphology (teratozoospermia), sperm motility parameters (asthenozoospermia), or sperm number (oligozoospermia or azoospermia) in human [12].

The cation channel of sperm associated *(CATSPER1*) gene is encodes an ion channel that involved in calcium (Ca^2+^) transport and play an important role in sperm motility [13]. Recently, association of *CATSPER1* gene variants with male infertility was reported [14]. The insertion mutations in this gene can cause to reduced fertility through sperm defects and asthenozoospermia [15]. In addition, other sperm defects such as low sperm count, reduced semen volume, non-motile sperm or reduced sperm motility, and increased structural abnormality in spermatozoa are reported in infertile males with *CATSPER1* gene mutation [14].

The spermatogenesis associated (*SPATA16*) gene is encodes a protein located in Golgi apparatus and pro-acrosomic vesicles which is associated with globozoospermia [16]. This gene plays an important role in spermatogenesis and sperm production with a major role in development of testis. Expression of *SPATA16* gene is extremely upregulated in human testes during puberty [17]. Several mutations and SNPs on this gene are reported in patients with non-syndromic monogenic male infertility [18].

The testis expressed (*TEX11*) gene is encodes a tetratricopeptide repeat motif that located in cytoplasm and spermatogonia nuclei type B and involved in protein–protein interactions in human [19]. This gene is exclusively expressed in human testis with the highest level in the zygotene phase of spermatocytes, and a basal level in late pachytene phase of spermatocytes [20, 21]. High expression of *TEX11* gene in early spermatocytes and spermatogonia type B indicate that *TEX11* gene plays a critical role in early stage of germ cells development [21].

To date, association of *CATSPER1* (rs2845570), *SPATA16* (rs1515442), and *TEX11* (rs143246552) genes polymorphism and male infertility was not investigated in Iranian Azeri population. Therefore, we investigated association of these polymorphisms and idiopathic male infertility in Iranian Azeri patients with idiopathic azoospermia and oligospermia.

## Methods

### Study subjects

In this case–control study, we recruited 200 Iranian Azeri men (25–50 years old) who were referred to Department of Infertility, Valiasr Hospital, Tabriz, Iran, during January 2018–December 2020. The case group consisted of 100 infertile males with confirmed idiopathic azoospermia and oligospermia using semen analysis. The control group consisted of 100 healthy fertile males without any abnormal sperm. The infertile males with ejaculatory duct obstruction, hypogonadotropic, orchitis, hypogonadism, cryptorchidism, as well as abnormal karyotype and/or microdeletions on Y chromosome were excluded from study. The characteristics and demographic variables of all subjects, (age, body mass index-BMI, alcohol drinking, tobacco smoking, family history of azoospermia and/or oligospermia, and semen parameters) were collected by questionnaires and interviews (Table 1). To prevent epidemiological bias, all studied subjects were recruited from the East Azerbaijan province, Iran. All participants were matched for age, races, and ethnic, as well as were genetically unrelated. This study was confirmed by ethical committee of Islamic Azad University, North Tehran Branch, Tehran, Iran (The ethical code: IR.IAU.TNB.REC.1399.030). The subjects were informed about the study, and signed a consent form according to the Declaration of Helsinki ethical standards.

**Table 1:** The demographic variables and characteristics of the studied patients and healthy control

Variables	Patients (n = 100)	Controls (n = 100)	*p* Value
Age (year ± SD)	29.33 ± 2.78	27.6 ± 3.06	0.298
BMI (kg/m ± SD)	26.25 ± 2.18	26.48 ± 2.34	0.699
*Tobacco smoking*			
Never	76 (76%)	89 (89%)	–
Ever	34 (34%)	11 (11%)	0.004*
*Alcohol drinking*			
Never	39 (39%)	31 (31%)	–
Ever	61 (61%)	69 (69%)	0.376
*Family history*			
Negative	79 (79%)	100 (100%)	–
Positive	21 (21%)	0 (0%)	0.008*
*Semen parameters*			
Concentration (× 106/ml)	Median: 3.5 (0–6.37)	125.5 (94–156.3)	< 0.0001*
Mann Whitney test	Mean: 3.71 ± 3.94	126 ± 40.3	
Motility (%)	Median: 48.5 (0–63)	60 (49–70)	< 0.0001*
Mann Whitney test	Mean: 33.95 ± 30.48	59.6 ± 11.55	
Volume (ml)	Median: 3.5 (2.35–4)	4 (3–5)	< 0.0001*
Mann Whitney test	Mean: 3.23 ± 1	4.18 ± 3.19	

*Statistically Significant *p* < 0.05, *BMI* body mass index

### DNA extraction

The peripheral blood sample (3 ml) were received from all patients and healthy controls, and collected into vials containing Ethylene diamine tetraacetic acid (EDTA) as an anticoagulant. The proteinase K method was employed to extraction of genomic DNA from collected peripheral blood samples. The nanodrop instrument was used to confirm quantity of the extracted DNA samples according to OD 260/280 ratio (desirable ratio was 1.7–1.9). Also, agarose gel electrophoresis was used to confirm quality of the extracted DNA samples. The genomic DNA samples with acceptable quantity and quality were stored at − 20 °C until genotyping.

### Primer design and synthesis

The reference sequences of *CATSPER1*, *SPATA16* and *TEX11* genes was obtained from National Center for Biotechnology Information (NCBI) online database (https://www.ncbi.nlm.nih.gov/gene). The primers design was conducted using Primer3 software for detection of *CATSPER1* (rs2845570), *SPATA16* (rs1515442) and *TEX11* (rs143246552) genes polymorphism. The sequences of the designed primers were sent to SinaClon Company, Iran for synthesis. The characteristics and sequences of the synthesized primers are presented in Table 2.

**Table 2:** The characteristics and sequences of primers used for detection of genes polymorphisms

Gene (polymorphism)	Primer sequence (5′ → 3′)	Products size: allele type
CATSPER1 (rs2845570)	Forward outer: TCCAGCATGACGGTGTTGAGGCAGA Reverse outer: ATATTCTTCTGCATCTACGTGGTGG Forward inner: TTCTTGCGGGTCCGCTGGAGCCG Reverse inner: TCTGGCCCTGTTCCTTTCAGCCGGCA	323 bp: allele G (A) 473 bp: – 198 bp: allele T (B)
SPATA16 (rs1515442)	Forward outer: TAACATCCTGGAAATGTCACAAGAG Reverse outer: TTCTTTAATCCCATACCTCAAGTGC Forward inner: ATGAAGTTGGTCTACATTGATGAAA Reverse inner: CTACAAACTCATAGCGAACACCCAC	256 bp: allele T (A) 515 bp: – 308 bp: allele C (B)
TEX11 (rs143246552)	Forward outer: ATAGATTCCAATCAGCATTAGTAACATC Reverse outer: AGGATTCAATATTTTCTCCAATATTCCC Forward inner: TCACTCTCAACAGTAATCTTCTCCAT Reverse inner: CCCTGAGGCTGACTTGACCG	348 bp: allele T (A) 563 bp: – 470 bp: allele C (B)

### DNA genotyping

Genotyping analysis of the obtained DNA samples from patients and control group was performed using tetra-primer amplification refractory mutation system-polymerase chain reaction (Tetra-ARMS-PCR) method and specific primers (Table 2) for *CATSPER1* (rs2845570), *SPATA16* (rs1515442) and *TEX11* (rs143246552) genes polymorphism. PCR amplification was conducted in a 25 μL total volume: template DNA (50 ng), forward primer (250 nM), reverse primer (250 nM), PCR Master Mix (1×). The condition of PCR reaction was as following: 1 cycle initial denaturation for 5 min in 94 °C, 30 cycles denaturation for 5 min in 94 °C, 30 cycles annealing for 45 s, 30 cycles extension for 45 s in 72 °C, 1 cycle final extension for 5 min in 72 °C. The obtained PCR products were electrophoresed on 2% agarose gel, and DNA bands size were estimated by a 50 bp marker (ladder). Presence of each alleles was detected according to bands size as showed in Table 2.

### Statistical analysis

The obtained data were analyzed using statistical SPSS software (version 21.0). Association of *CATSPER1* (rs2845570), *SPATA16* (rs1515442) and *TEX11* (rs143246552) genes polymorphism with azoospermia and oligospermia were investigated using logistic regression. Fisher’s exact test and chi-square (χ^2^) test were used to analysis of Hardy–Weinberg equilibrium (HWE) in genotypes distribution of case and control groups. Differences in demographic variables and characteristics of the studied infertile patients and healthy controls were analyzed by independent sample t-test. The statistically significant was considered as *p* < 0.05.

## Results

### Demographic characteristics

The demographic variables and characteristics of patients and healthy controls are presented in Table 1. The statistical analysis demonstrated that there were no significant differences between patients and healthy controls in terms of alcohol drinking, body mass index (BMI), and age (*p* > 0.05); whereas we find a significant difference between patients and healthy controls in term of positive family history of infertility and tobacco smoking (*p* < 0.05). These results indicated that the positive family history and tobacco smoking can cause to increase male azoospermia and oligospermia risk.

### Hardy–Weinberg equilibrium

The statistical analysis indicated that the genotype distribution of *CATSPER1* (rs2845570), *SPATA16* (rs1515442) and *TEX11* (rs143246552) genes polymorphism in patients and healthy controls were in agreement with HWE (*p* > 0.05).

### Genotype and allele distribution

The genotype and allele distribution of patients and healthy controls are presented in Table 3. Our study indicated a significant correlation between *CATSPER1* (rs2845570) and *SPATA16* (rs1515442) genes polymorphisms and idiopathic azoospermia and oligospermia; whereas, no correlation was found between *TEX11* (rs143246552) gene polymorphism and idiopathic azoospermia and oligospermia (Table 3).

**Table 3:** Genotype and allele frequencies of the studied polymorphisms in patients and healthy control

Gene (polymorphism)	Inheritance models	Genotype and Allele	Case (%)	Control (%)	*p* Value	OR (95% CI)
CATSPER1 (rs2845570)	Codominant	GG	81 (81%)	98 (98%)	Ref	Ref = 1
		GT	14 (14%)	2 (2%)	0.001*	8.47 (2.14–38.1)
		TT	5 (5%)	0 (0%)	0.021*	Infinity (1.7–infinity)
	Dominant	GG	81 (81%)	98 (98%)	Ref	Ref = 1
		GT + GG	19 (19%)	2 (2%)	0.0001*	11.49 (2.8–50.7)
	Recessive	TT	5 (5%)	0 (0%)	Ref	Ref = 1
		GT + GG	95 (95%)	100 (100%)	0.059	Infinity (1.5–infinity)
	Overdominant	GT	14 (14%)	2 (2%)	Ref	Ref = 1
		GG + TT	86 (86%)	98 (98%)	0.0029*	7.97 (2.02–35.8)
	Alleles	G wild	176 (88%)	198 (99%)	Ref	Ref = 1
		T mutant	24 (12%)	2 (1%)	0.0001*	13.5 (3.6–58.4)
SPATA16 (rs1515442)	Codominant	TT	87 (87%)	96 (96%)	Ref	Ref = 1
		TC	12 (12%)	4 (4%)	0.039*	3.31 (1.01–9.61)
		CC	1 (1%)	0 (0%)	0.478	Infinity (0.12–infinity)
	Dominant	TT	87 (87%)	96 (96%)	Ref	Ref = 1
		TC + CC	13 (13%)	4 (4%)	0.039*	3.58 (1.14–10.3)
	Recessive	CC	1 (1%)	0 (1%)	Ref	Ref = 1
		TC + TT	99 (99%)	100 (100%)	> 0.999	Infinity (0.11–infinity)
	Overdominant	TC	12 (12%)	4 (4%)	Ref	Ref = 1
		CC + TT	88 (88%)	96 (96%)	0.065	3.27 (1.0–9.5)
	Alleles	T wild	186 (93%)	196 (98%)	Ref	Ref = 1
		C mutant	14 (7%)	4 (2%)	0.016*	3.69 (1.2–10.4)
TEX11 (rs143246552)	Codominant	TT	95 (95%)	98 (98%)	Ref	Ref = 1
		TC	2 (2%)	2 (2%)	0.999	1.03 (0.815–6.7)
		CC	3 (3%)	0 (0%)	0.246	Infinity (0.87–infinity)
	Dominant	TT	95 (95%)	98 (98%)	Ref	Ref = 1
		TC + CC	5 (5%)	2 (2%)	0.444	2.58 (0.53–13.1)
	Recessive	CC	3 (3%)	0 (0%)	Ref	Ref = 1
		TC + TT	97 (97%)	100 (100%)	0.246	Infinity (0.87–infinity)
	Overdominant	TC	2 (2%)	2 (2%)	Ref	Ref = 1
		CC + TT	98 (98%)	98 (98%)	1	1 (0.15–6.48)
	Alleles	T wild	192 (96%)	198 (99%)	Ref	Ref = 1
		C mutant	8 (4%)	2 (1%)	0.055	4.12 (1.0–19.4)

*Statistically Significant *p* < 0.05

*OR* odds ratio, *CI* confidence interval

In the *CATSPER1* (rs2845570) gene polymorphism, frequency of wild homozygous (GG), heterozygous (GT), and mutant homozygous (TT) genotypes in case group are 81%, 14% and 5%, respectively. Whereas, frequency of GG, GT, and TT genotypes in case group are 98%, 2% and 0%, respectively. The obtained results demonstrated a significant increased risk of idiopathic azoospermia and oligospermia in patients with AB (*p* = 0.001; OR = 8.47; 95% CI: 2.14–38.1) and BB (*p* = 0.021; OR = infinity; 95% CI: 1.7-infinity) genotypes. We suggested that presence of B mutant allele can cause to significantly increase in idiopathic azoospermia and oligospermia (Table 3).

In the *SPATA16* (rs1515442) gene polymorphism, frequency of wild homozygous (TT), heterozygous (TC), and mutant homozygous (CC) genotypes in case group are 87%, 12% and 1%, respectively. Whereas, frequency of TT, TC, and CC genotypes in control group are 96%, 4% and 0%, respectively. We indicated that the heterozygote AB (*p* = 0.039; OR = 3.31; 95% CI 1.01–9.61) genotype can cause to a significant increase in risk of idiopathic azoospermia and oligospermia (Table 3).

In the *TEX11* (rs143246552) gene polymorphism, frequency of wild homozygous (TT), heterozygous (TC), and mutant homozygous (CC) genotypes case group are 95%, 2% and 3%, respectively. Whereas, frequency of TT, TC, and CC genotypes in control group are 98%, 2% and 0%, respectively. We did not find any significant correlation between *TEX11* (rs143246552) gene polymorphism and idiopathic azoospermia and oligospermia (Table 3).

## Discussion

Infertility is defined as the inability to conceive after at least twelve months of regular and unprotected intercourse [22]. Idiopathic azoospermia and oligospermia are the common type of male infertility, which has recently become a research focus. The azoospermia and oligospermia etiology are includes abnormal immunity, infection, endocrine dysfunction, abnormal semen liquefaction, chromosomal abnormalities, varicocele, and abnormal sperm structure. Idiopathic azoospermia and oligospermia, with complex molecular mechanisms, can cause to abnormal sperm quantity that closely associated with abnormal fertilization [23, 24]. In the recent years, extensive studies have been performed to identify of molecular mechanisms of male infertility; however, a large proportion of male infertility cases are idiopathic. Evidence suggested that the common genetic causes of male azoospermia or oligospermia are related to mutations and SNPs on involved genes in spermatogenesis [7, 25].

In the present study, we recruited 100 patients with idiopathic male infertility (azoospermia and/or oligospermia) and 100 healthy controls from Iranian Azeri population in order to investigate correlation of *CATSPER1* (rs2845570), *SPATA16* (rs1515442), and *TEX11* (rs143246552) genes polymorphism with idiopathic azoospermia and oligospermia risk. We demonstrated a correlation between *CATSPER1* (rs2845570) and *SPATA16* (rs1515442) genes polymorphisms with idiopathic azoospermia and oligospermia. However, no correlation was observed between *TEX11* (rs143246552) gene polymorphism and idiopathic azoospermia and oligospermia.

Previous studies have demonstrated that *CATSPER1* gene is strongly associated with sperm motility as well as acrosome reaction and sperm hyper-activation [26, 27]. Moreover, clinical studies have reported that abnormal expression of *CATSPER1* gene are associated with idiopathic infertility patients with decreased sperm motility [28, 29]. So far, only two studies have been investigated association of *CATSPER1* gene polymorphisms with idiopathic male infertility. In a study by Rahimpour Goushchi et al. [30] reported that the frequency of the CC allele in *CATSPER1* rs1893316 polymorphism significantly increased in patients with male infertility in Iranian Azeri population. In another study by Shu et al. [31] reported that there is no significant association between *CATSPER1* rs2845570 polymorphism and male infertility in Chinese population. To our knowledge, the present study is the first report on significant association of *CATSPER1* rs2845570 polymorphism and male infertility in the world.

Recently, studies have suggested that *SPATA16* gene is involved in sperm production and can cause to globozoospermia, testicular diseases, spermatogenesis arrest, and sperm aneuploidy in human [32, 33]. So far, very limited studies have been reported on *SPATA16* gene polymorphisms and male infertility. Therefore, the exact role of *SPATA16* gene polymorphisms are unknown in molecular pathogenesis of male infertility. In a study by Roozbahani et al. (2017) reported that there is no significant association between *SPATA16* rs137853118 exon 4 polymorphism and male infertility in Iranian population [32]. In another study by Dom et al. [18] suggested that the homozygous c.848G > A mutation in *SPATA16* gene can cause to male infertility with an autosomal recessive mode of transmission in three patients from a Jewish family. To our knowledge, this study is the first description in association of *SPATA16* (rs1515442) genes polymorphism and male infertility in the world.

Evidence indicated that *TEX11* gene is involved in chromosomal synapsis and meiotic recombination and can cause to oligozoospermia, azoospermia, meiotic arrest, and male infertility [34, 35]. In recent years, two studies extensively investigated *TEX11* gene polymorphisms correlation with male infertility [36, 37]. In a study by Sezavar et al. [36] reported that rs6525433 polymorphism in *TEX11* gene were not associated with risk of azoospermia in Iranian patients with infertility. In another study by Zhang et al. [37] indicated that there is a significant association between *TEX11* rs6525433 polymorphism and male infertility in Chinese population; whereas they found no significant association between *TEX11* rs4844247 polymorphism and male infertility in this population. However, no study has been reported on effect of *TEX11* rs143246552 polymorphism on male infertility. To our knowledge, our study is the first report on association lack of *TEX11* rs143246552 polymorphism and male infertility in the world.

Results contradiction reported by different studies might be due to other involved genes SNPs that cause male infertility, differences in geographic area, sample size and sample selection bias, genetic background of population and heterogeneity in ethnicity and race, and environmental factors [38, 39].

## Conclusions

In general, our study provided a more understanding of male infertility as a heterogeneous disorder, and suggested that *CATSPER1* (rs2845570) and *SPATA16* (rs1515442) genes polymorphism are significantly associated with the risk of idiopathic azoospermia and oligospermia in Iranian Azeri population. In addition, we found no significant association between *SPATA16* (rs1515442) gene polymorphism and idiopathic azoospermia and oligospermia in Iranian Azeri population. Therefore, to access more accurate results in association of these polymorphisms with idiopathic azoospermia and oligospermia, more extensive studies are recommended with larger sample size on other populations, ethnic origins, and races.

## Data Availability

The datasets generated during the current study are available in the [zenodo] repository, Accession No.: 5885267 (https://zenodo.org/record/5885267).

## Abbreviations

SNP: Single nucleotide polymorphism
Tetra-ARMS-PCR: Tetra-primer amplification refractory mutation system-polymerase Chain reaction
EDTA: Ethylene diamine tetraacetic acid
SPSS: Statistical package for the social sciences
HWE: Hardy–Weinberg equilibrium
OR: Odds ratios
CI: Confidence interval
BMI: Body mass index
IVF: In vitro fertilization
NCBI: National center for biotechnology information
